# Evaluation of early body shape for epidemiological research in absence of objective measurements

**DOI:** 10.1186/1753-6561-6-S3-P70

**Published:** 2012-06-27

**Authors:** Sally N Akarolo-Anthony, Clement A Adebamowo

**Affiliations:** 1 Nutrition Department, Harvard School of Public Health, Boston, MA, 02215, USA; 2 Epidemiology and Public Health, School of Medicine, University of Maryland, Baltimore, MD, 021201, USA

## Background

Developing countries are undergoing epidemiological transitions with increasing prevalence of non-communicable diseases (NCD). There is increasing appreciation of the role of early life exposure in the etiology of many of these conditions particularly the role of body shape and size for given age. However, many of these environments lack early life records of birth and growth. So it is not possible to correlate early life characteristics with risk of disease in these populations. In this study we, evaluated the use of body images as an estimator of body size and characteristics.

## Methods

1058 workers at a government office in Nigeria were enrolled in a study of body size, dietary energy intake and physical activity. The participants answered an interviewed administered questionnaire who also measured their anthropometric characteristics.

## Results

The mean age (SD) of participants was 41.5 (9.3) years and mean BMI (SD) was 27.0 (4.9), mean waist (SD) = 75.4 (26.8) cm, mean hip circumference (SD) = 86.7 (33.1) cm, mean waist hip ratio (WHR) = 0.88 (0.10) and mean weight (SD) = 74.6 (14.2) kg. There was strong correlation between the interviewee perception of their current body type and that of the interviewer (r = 0.73, p-value < 0.001). Compared with anthropometric characteristics, the body images correlated most strongly with BMI (r = 0.56, p-value < 0.001) but less so with waist circumference (r = 0.33), hip circumference (r = 0.33), WHR (r = 0.15).

## Conclusions

Our results suggest that study participants are able to evaluate their current body shape and correlate with it images. These images correlate well with BMI and can be used in epidemiological studies where availability of objective measures are limited. The study is now being extended to evaluate body shape in early life.

